# The *Early Elementary School Abbreviated Math Anxiety Scale* (the EES-AMAS): A New Adapted Version of the AMAS to Measure Math Anxiety in Young Children

**DOI:** 10.3389/fpsyg.2020.01014

**Published:** 2020-05-21

**Authors:** Caterina Primi, Maria A. Donati, Viola A. Izzo, Veronica Guardabassi, Patrick A. O’Connor, Carlo Tomasetto, Kinga Morsanyi

**Affiliations:** ^1^NEUROFARBA, University of Florence, Florence, Italy; ^2^Department of Developmental and Social Psychology, Sapienza University of Rome, Rome, Italy; ^3^Department of Psychology, University of Bologna, Bologna, Italy; ^4^School of Psychology, Queen’s University, Belfast, United Kingdom

**Keywords:** AMAS, early elementary school children, confirmatory factor analysis, invariance, gender differences, math anxiety, reliability, validity

## Abstract

In the past decade, there has been increasing interest in understanding how and when math anxiety (MA) develops. The incidence and effects of MA in primary school children, and its relations with math achievement, have been investigated. Nevertheless, only a few studies have focused on the first years of primary school, highlighting that initial signs of MA may emerge as early as 6 years of age. Nevertheless, there are some issues with measuring MA in young children. One of these is that, although several scales have been recently developed for this age group, the psychometric properties of most of these instruments have not been adequately tested. There is also no agreement in the number and identity of the factors that underlie MA at this young age. Some scales also consist of several items, which make them impractical to use in multivariate studies, which aim at the simultaneous measurement of several constructs. Finally, most scales have been developed and validated in US populations, and it is unclear if they are appropriate to be used in other countries. In order to address these issues, the current studies aimed at developing a short, new instrument to assess MA in early elementary school students, the *Early Elementary School Abbreviated Math Anxiety Scale* (the EES-AMAS). This scale is an adapted version of the *Abbreviated Math Anxiety Scale* (AMAS; Hopko et al., 2003), which is one of the most commonly used scales to measure MA and has been shown to be a valid and reliable measure across a number of countries and age groups. The psychometric properties of the new scale have been investigated by taking into account its dimensionality, reliability, and validity. Moreover, the gender invariance of the scale has been verified by showing the measurement equivalence of the scale when administered to male and female pupils. We have also demonstrated the equivalence of the scale across languages (Italian and English). Overall, the findings confirmed the validity and reliability of the new scale in assessing the early signs of math anxiety and in measuring differences between genders and educational contexts. We have also shown that MA was already related to math performance, and teacher’s ratings of children’s math ability at this young age. Additionally, we have found no gender differences in MA in our samples of 6- and 7-year-old children, an important finding, given the strong evidence for gender differences in MA in older age groups.

## Introduction

Although mathematical proficiency is becoming increasingly important, especially in technological societies, it has been estimated that about 17% of the population (Luttenberger et al., 2018) suffer from more or less severe psychological or physiological symptoms related to feelings of anxiety when confronted with tasks that require the use of numerical information. Data from the Programme for the International Student Assessment (PISA), which tests 15-year-old students, reported that 31% stated that they get very nervous when they do math problems (Organisation for Economic Co-operation and Development, 2013). Math anxiety (MA) has been described as a feeling of tension and anxiety that interferes with the manipulation of numbers in a wide variety of ordinary life and academic situations (Richardson and Suinn, 1972), and it represents an obstacle to mathematical development.

MA has been found to have a negative relationship with mathematics performance and achievement (Hembree, 1990; Ma, 1999). Researchers have reported a consistent, weak to medium negative relationship between math anxiety and performance (ranging from −0.11 to −0.36) indicating that students with higher levels of MA tend to show poorer mathematics performance. Data from the PISA studies confirm these results within and across countries (Organisation for Economic Co-operation and Development, 2013). Additionally, MA may have a number of important indirect effects. Highly math anxious students participate less in math lessons and enjoy them less, they perceive their mathematical abilities to be poorer and are less likely to see the value of learning math (e.g., Hembree, 1990; Ma, 1999). A particularly problematic consequence of MA is that individuals with higher level of anxiety tend to avoid taking high school and college or university mathematics courses. Indeed, similar to other performance-based anxieties, MA involves psychological arousal, negative cognitions, escape and/or avoidance behaviors and, when the individual cannot avoid the situation, performance deficits. MA is also related to reduced cognitive reflection (Morsanyi et al., 2014; Primi et al., 2018), and poorer decision making performance (e.g., Rolison et al., 2016; Rolison et al., 2020).

In the past decade, there has been increasing interest in understanding how and when MA develops (Wu et al., 2012; Harari et al., 2013; Jameson, 2013; Ramirez et al., 2013; Dowker et al., 2016). Studies have investigated the incidence and effects of MA in primary school samples (e.g., Karasel et al., 2010; Galla and Wood, 2012; Wu et al., 2012), and its relation to math achievement (Ramirez et al., 2016). However, only a few studies have focused on younger pupils, although initial signs of MA may emerge as early as 6 years of age (Aarnos and Perkkilä, 2012), and MA has important implications for later development, as it appears fairly stable over time (Ma and Xu, 2004; Krinzinger et al., 2009; Cargnelutti et al., 2017).

## The Assessment of Math Anxiety in Early Primary School

One of the reasons why it is difficult to conduct research into MA in younger children relates to the assessment of MA (see Cipora et al., 2019). Following the first scale, which was developed to exclusively investigate MA, the *Mathematical Anxiety Rating Scale* – MARS (Richardson and Suinn, 1972), a substantial number of scales have been created. These scales vary in their target population, length, and psychometric properties. In fact, the psychometric properties of many of these scales have not been adequately tested. Limitations include small sample sizes, the weakness of validity data, the lack of test-retest analyses, as well as the lack of confirmatory procedures to assess the dimensionality of the scales, and the absence of normative data (Eden et al., 2013; Harari et al., 2013). Additionally, instruments for children have mostly been adapted from scales for adults and/or have been developed for samples with a limited age range. Finally, cross-national investigations of the psychometric properties of these scales are also lacking.

Focusing on the already existing instruments for younger children (see Table 1), we have prepared an overview of the psychometric properties of these scales. First, we have found that the interest in assessing MA in younger children has only emerged recently. Indeed, all papers regarding the psychometric properties of these scales have been published after 2010. Additionally, among the seven included instruments, only the *Children’s Anxiety in Math Scale* (CAMS; Jameson, 2013) and the *Mathematics Anxiety Questionnaire* (MAQ), originally developed by Thomas and Dowker (2000) and examined by Wood et al. (2012) were completely newly developed, whereas the other scales (i.e., the *Mathematics Anxiety Rating Scale for Elementary School Children;* MARS-E; Suinn et al., 1988; the *Mathematics Anxiety Questionnaire;* MAQ; Wigfield and Meece, 1988; and the *Mathematics Anxiety Scale for Children;* MASC; Chiu and Henry, 1990; the *Child Math Anxiety Questionnaire* (CMAQ; Ramirez et al., 2013) and the *Mathematics Anxiety Scale for younger children* (MASYC; Harari et al., 2013) have been developed from an already existing tool, the MARS (Richardson and Suinn, 1972). Finally, two scales are revised versions of previously developed instruments for children: the *Child Math Anxiety Questionnaire Revised* (CMAQ-R; Ramirez et al., 2016) and the *Revised Mathematics Anxiety Scale for younger children* (MASYC-R; Ganley and McGraw, 2016).

**TABLE 1:** Psychometric properties of the math anxiety scales for early elementary school children.

**Instrument**	**Study**	**Newly developed**	**N items**	**Dimensionality**	**Realiabiity**	**Validity**	**Invariance across genders**	**Language**
CAMS	Jameson, 2013	Yes	16	EFA	α = 0.86	Face criterion	No	English
Child Math Anxiety Questionnaire (CMAQ)	Ramirez et al., 2013	MARS-E (Suinn et al., 1988)	8	No	α = 0.55	No	No	English
Child Math Anxiety Questionnaire-Revised (CMAQ-R)	Ramirez et al., 2016	CMAQ (Ramirez et al., 2013)	16	No	α = 0.83	No	No	English
Math Anxiety Questionnaire (MAQ)	Wood et al., 2012	yes	24	Mokken automatic item classification Multidimensional scaling	α = 0.87	Predictive	No	English Portuguese German
Mathematics Anxiety Scale for Young children (MASYC)	Harari et al., 2013	MARS-E (Suinn et al., 1988) MAQ (Wigfield and Meece, 1988)	12	EFA	F1 (α = 0.70) F2 (α = 0.72) F3 (α = 0.67)	Criterion Predictive Gender differences	No	English
Revised Mathematics Anxiety Scale for Young children (MASYC-R)	Ganley and McGraw, 2016	MASYC (Harari et al., 2013)	13	CFA	α = 0.87	Criterion Convergent (MASYC) Gender differences	No	English
Scale for Early Mathematics Anxiety (SEMA)	Wu et al., 2012	MARS-E (Suinn et al., 1988) MASC (Chiu and Henry, 1990)	20	EFA	α = 0.87 Split-half = 0.77	Criterion	No	English

Concerning the psychometric properties of these scales, information regarding dimensionality has been provided for all scales, except for the CMAQ (Ramirez et al., 2013) and the CMAQ-R (Ramirez et al., 2016). In the case of three scales, the CAMS, the MASYC, and the *Scale for Early Mathematics Anxiety* (SEMA; Wu et al., 2012), dimensionality has been tested using Exploratory Factor Analysis (EFA), whereas in the case of the MAQ, a multidimensional scaling procedure has been used. There is only one scale (the MASYC-R) where dimensionality has been investigated using Confirmatory Factor Analysis (CFA). Overall, all of these studies showed that MA, even at a young age, is a multidimensional construct. Nevertheless, the number of factors have varied between two and four, and the identity of these factors have also differed between the scales. Concerning the CAMS, EFA has identified three factors, namely General Math Anxiety, Math Performance Anxiety, and Math Error Anxiety; whereas the MAQ consists of four factors (i.e., Self- Perceived Performance, Attitudes in Mathematics, Unhappiness Related to Problems in Mathematics and Anxiety Related to Problems in Mathematics); although multidimensional scaling suggested that these may be combined into two factors (i.e., Self-perceived performance and attitudes, resulting from the combination of the first two factors, and Mathematics Anxiety, resulting from the combination of the other two factors). Moreover, both the MASYC and the MASYC-R have three factors (i.e., Negative Reactions, Numerical Confidence, and Worry). Finally, the SEMA includes two correlated factors: Numerical Processing Anxiety and Situational and Performance Anxiety.

Concerning the reliability of the scales, this has been measured as internal consistency and reliability indices have been provided for all scales. Additionally, Wu et al. (2012) also provided split-half reliability. Following the cut-off criteria for internal consistency proposed by the European Federation of Psychologists’ Associations (Evers et al., 2013), values range from moderate to high for all scales, except for the CMAQ, which is the shortest scale with only eight items, for which Cronbach’s alpha was 0.55. Indeed, Cronbach’s alfa is strongly influenced by the number of items. Nevertheless, scales for early elementary school students must be short, otherwise children get fatigued.

Validity measures have been provided by all studies, although the specific types of validity that were examined varied across studies. Face validity has been considered only by Jameson’s study (2013), as items were independently reviewed by five experts who confirmed the appropriateness of the items.

Criterion validity, which examines the relations between math anxiety and other related constructs, has mostly been investigated in relation to math achievement, and it has been reported for the CAMS, the MASYC, the MASYC-R, and the SEMA. Additionally, it has been investigated in relation to trait and general anxiety (for the SEMA and the MASYC-R, respectively), math reasoning (for the SEMA), and math confidence, math interest and math importance (for the MASYC-R). The relations with computation and counting skills, math concepts and attitude toward mathematics have been investigated for the MASYC (Harari et al., 2013). Moreover, to identify the best predictors of MA, a regression analysis was conducted by Harari et al. (2013), which included general anxiety, math performance and math attitudes. Results regarding the MASYC- R suggest that a substantial proportion of the variance in MA is explained by these variables. Additionally, to investigate the predictive validity of the MAQ, regression analyses entering the four MAQ subscales as predictors of numeric and arithmetic abilities were conducted. Results showed that the “Self-perceived Performance” subscale was a significant predictor of basic and complex arithmetic abilities even after controlling gender, age and verbal and nonverbal short-term memory. Concerning convergent validity, the correlation between instruments that assess the same construct was only reported between the MASYC and the MASYC-R. Our review of the literature has also shown the overall absence of investigations regarding measurement invariance across genders, although gender differences in MA are commonly investigated (Eden et al., 2013; Harari et al., 2013). When studying test invariance, we determine whether a tool functions equivalently in different groups, that is, we test the absence of biases in the measurement process. In other words, the observed scores should depend only on the latent construct, and not on group membership. An observed score is said to measure the construct invariantly, if it depends on the true level of the trait in a specific person, rather than on group membership or context (Meredith, 1993). This means that people belonging to different groups, but with the same level of a trait, are usually expected to display similar response patterns on items that measure the same construct. Unfortunately, the gender invariance of the commonly used measurement tools in the MA literature has not been investigated. Another limitation is the absence of different language versions of the scales. Only one scale (the MAQ) has German and Portuguese versions available; all the other scales only have an English version.

In sum, the psychometric properties of these scales have been, in general, inadequately tested, due to the lack of confirmatory procedures to assess the dimensionality of the scales, and because inadequate measures of validity and reliability were used. In particular, convergent validity has only been investigated in the case of a few scales. The invariance of the scales across genders and languages has also not been confirmed, which makes group comparisons ambiguous, because it makes it difficult to tell whether any group differences are a function of the trait being measured, or artifacts of the measurement process (Vandenberg and Lance, 2000).

## The Development of the *Early Elementary School Students – Abbreviated Math Anxiety Scale* (EES-AMAS)

Starting from these premises, the current work was aimed at developing a new instrument to assess MA in early elementary school students, overcoming some of the limitations of the currently available scales and with the advantage of being short (Widaman et al., 2011). Among the measures of MA used with adults but also recently adapted for children between the ages of 8–11 (Italian version by Caviola et al., 2017) and 8–13 (English version by Carey et al., 2017), the AMAS (*Abbreviated Math Anxiety Scale*; Hopko et al., 2003) has presented this property with only nine items. It was originally developed using the highest loading items from the MA Rating Scale (MARS; Richardson and Suinn, 1972) and it is considered a parsimonious, reliable, and valid scale for assessing MA, with two factors: Learning Math Anxiety, which relates to anxiety about the process of learning, and Math Evaluation Anxiety, which is more closely related to testing situations. Indeed, it is one of the most commonly used tools to measure MA in college and high school students (for a review, see Eden et al., 2013). It has been translated into several languages, including Polish (Cipora et al., 2015, 2018), Italian (Primi et al., 2014), Persian (Vahedi and Farrokhi, 2011) and German (Dietrich et al., 2015; Schillinger et al., 2018). These translations have been found to be valid and reliable, confirming the cross-cultural applicability of the AMAS.

For these reasons, the AMAS has been chosen as the starting point for developing our instrument, the *Early Elementary School Students – Abbreviated Math Anxiety Scale* (EES-AMAS), with the aim of also maintaining the two-dimensional structure of the original scale. The adaptation mainly concerned the need to make the scale suitable for young children. Indeed, age-appropriate vocabulary was considered a priority to maximize the comprehensibility of the scale (Ganley and McGraw, 2016). This has been achieved by modifying, when necessary, the content of the items to ensure understanding (i.e., by using simple and familiar words). Additionally, the age-appropriateness and meaningfulness of the content has also been ensured by creating items which were consistent with children’s study habits, mathematics course organization and materials. For example, one of the original items of the Learning Math Anxiety factor was “*Having to use the tables in the back of a math book.*” This has been changed to: “*When you are using the Number Line*” One of the original items of the Evaluation Math Anxiety factor was: “*Being given a “pop” quiz in math class.*” This has been changed to: “*When your math teacher asks you to solve a maths sum.*”

Subject matter experts (teachers and developmental psychologists) have been asked to evaluate whether the test items assess the intended content and if they are suitable for children. Inter-rater reliability indices (Cohen’s Kappa) have been used to measure the agreement between raters, and adjustments have been made to obtain the final version of the EES -AMAS.

**FIGURE 1:**
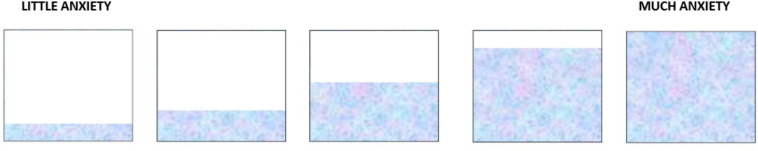
The rating scale used to measure the level of anxiety elicited by each situation described by the items of the EES-AMAS. Children had to respond by pointing at the appropriate box.

Additionally, the response scale has been modified to suit the target age group. Instead of using a Likert scale with numbers, we have used a pictorial scale, in line with other studies (e.g., Thomas and Dowker, 2000; Wu et al., 2012; Jameson, 2013). However, instead of using smiley faces that children could not interpret correctly (for example, some children assumed that they were expected to choose the face which was the most similar to them), we have created a pictorial scale using boxes (Figure 1). For each item that described a familiar behavior related to the learning or evaluation of math, participants were asked to choose the box with the level of anxiety (from little to much anxiety) that each statement evoked. We have used the word “anxiety” instead of “worry” (e.g., Thomas and Dowker, 2000) or feeling “nervous” (Wu et al., 2012), as teachers confirmed that children at this age were already familiar with the term “anxiety.”

In this study, using CFA, we expected to confirm the two-factors structure of the scale even at this young age. Several studies have found that MA, even at a young age, is a multidimensional construct (e.g., Wu et al., 2012; Harari et al., 2013; Jameson, 2013), although the number and identity of these factors differ across instruments. An advantage of adapting the same scale for different age groups is that it makes it easier, and more meaningful, to investigate developmental changes in MA.

Additionally, a short measure is more useful considering that MA is typically investigated together with other related constructs (e.g., math performance). However, it is also important to use scales that are reliable. The Cronbach alfa coefficient is widely used to estimate the reliability of MA. Nevertheless, using an inter-item correlation matrix may lead to an underestimation of reliability, especially when the scale contains a small number of items (Yang and Green, 2011). Indeed, as reported by Deng and Chan (2017), the application of coefficient alpha has been criticized (see, e.g., Green et al., 1977; Raykov, 1997; Sijtsma, 2009; Yang and Green, 2011). This is because, the sample coefficient alpha yields a consistent estimate of reliability only when all items have equal covariance with the true score (i.e., when item scores fit a unidimensional model in which the loadings are set to be equal and errors are uncorrelated). However, this assumption is seldom met in practice by educational and psychological scales (see, e.g., Lord and Novick, 1968; Jöreskog, 1971; Green and Yang, 2009). A measure that overcomes the issues with alpha is coefficient omega (ω) (McDonald, 1978). It is defined as the ratio between the variance due to the common factor and the variance of the total scale scores. In the current study, to overcome the limitations of the Cronbach’s alfa coefficient, we measured the reliability of the EES-AMAS using omega. However, to make it easier to compare the reliability of our scale with other versions of the AMAS, we also report alpha and ordinal alpha (based on polychoric correlations instead of the typical Pearson coefficients), which were used as alternative indices of reliability in previous studies (e.g., Cipora et al., 2015; Pletzer et al., 2016; Carey et al., 2017; Devine et al., 2018).

There is a large body of literature examining whether there are gender differences in MA, but unfortunately the measurement tools that are often employed in research are not necessarily gender-invariant. If observed gender differences have been obtained by employing noninvariant scales across genders, the overall findings might be misleading because it is impossible to tell whether these differences reflect actual differences in MA among males and females or if they reflect differences related to group membership. In order to understand gender differences, it is important to employ instruments where invariance across genders has been verified. Thus, we aimed to test the invariance of the EES-AMAS across genders in young pupils.

Additionally, applying the same method, we also tested the equivalence of the EES-AMAS across languages (Italian versus British English). Testing the invariance of the test concerns the extent to which the psychometric properties of the test generalize across groups or conditions. Indeed, invariance ensures both the fairness and validity of group comparisons while examining a specific psychological construct (Kane, 2013). Therefore, measurement invariance is a prerequisite of the evaluation of substantive hypotheses regarding differences between contexts and groups.

Finally, we tested the validity of the scale by investigating the relations between MA and math achievement. Studies have mainly focused on secondary school and university students, and they have almost always found a negative relationship between these constructs (−0.18 < *r* < −0.48) (Luttenberger et al., 2018). By contrast, the few studies that were conducted with primary school samples have yielded contradictory results: some did not find a correlation (Thomas and Dowker, 2000), others have found that MA was negatively linked to math achievement (e.g., Wu et al., 2012). However, a limitation of comparing this relation across different studies is that they have used different measures to assess achievement (typically, scores on achievement tests or grades). In this study, to measure math performance, a similar test was developed and administered in the Italian and British samples.^1^ Additionally, to address the lack of measures of convergent validity, we have tested the relation of the EES-AMAS with another measure of MA developed for this age group, the CMAQ-R (Ramirez et al., 2016). Thus, we expected to find a negative correlation between MA and math achievement and a positive correlation between the two measures of MA in both samples.

In sum, in these studies, we have investigated the psychometric properties of the EES-AMAS, a new scale, which was developed with the purpose of overcoming some of the limitations of MA assessment in young children. In detail, in Study 1, with an Italian sample, we investigated the dimensionality of the scale using a confirmatory procedure, we measured the reliability of the scale with coefficient omega (ω) (McDonald, 1978), and its validity, measuring its relationship with math achievement. Moreover, we tested the invariance of the scale across genders. In Study 2, we investigated the invariance of the scale across languages (Italian and British English) and we tested the validity of the scale in both educational contexts, using measures of both criterion and convergent validity.

## Study 1

### Materials and Methods

#### Participants

The study involved 150 children (Mean age = 7.1 years; *SD* = 0.57; 57% female) attending Italian primary schools in central Italy; 73 (49%) were in grade 1 (Mean age = 6.6 years; *SD* = 0.26; 63% female) and 77 (51%) were in grade 2 (Mean age = 7.6 years; *SD* = 0.29; 51% female).

A detailed study protocol that explained the aims and methodology of the study was approved by the institutional review boards of the schools. Parental consent was obtained for all children before they took part in the study, which assured them that the data obtained would be handled confidentially and anonymously.

#### Materials and Procedure

The *Early Elementary School Students*-MAS (EES-AMAS) contains nine Likert-type items related to two aspects of math anxiety measured by the subscales: Learning Math Anxiety-LMA (5 items, for example “*When you are using the number line*”) and Math Evaluation Anxiety-MEA (4 items, for example,” *When your maths teacher asks you to solve a maths word problem*”). Participants responded to the items using a pictorial scale consisting of partially filled boxes with a varying level of content from “little” to “much” anxiety (rated 1–5) (Figure 1).

The scale was individually administered. A trained interviewer presented a brief description of anxiety with some examples (see Appendix) to each child, and explained the response scale with the boxes. After this preliminary introduction, each item was read aloud by the interviewer who recorded each answer that the participant gave by pointing at a box on the response sheet. It took about 10 min to complete the scale.

The AC-MT 6–11 (Cornoldi et al., 2012) was used to measure mathematics achievement. It is a standardized mathematics test designed for first- to fifth-graders to assess calculation procedures and number comprehension. In this study, participants had to solve 4 written multi-digit calculations (two additions, two subtractions) designed for first- and second-graders. The test was paper and pencil administered and it took about 10 min to complete. Both measures were administered individually during class time in a random order.

### Results

Item distributions and descriptives were examined to assess normality (Table 2). Skewness and kurtosis indices of some items revealed that the departures from normality were not acceptable (Marcoulides and Hershberger, 1997).

**TABLE 2:** Means, standard deviations (SDs), skewness, kurtosis, and item- total correlations for each item, and factor loadings of the EES-AMAS.

**Item**	**Mean (*SD*)**	***Skewness***	**Kurtosis**	**Corrected item-total correlations**	**LMA**	**EMA**
1	1.70 (1.34)	1.74	1.46	0.33	0.45	
3	2.11 (1.42)	0.98	–0.46	0.55	0.67	
6	2.54 (1.61)	0.44	–1.44	0.46	0.53	
7	2.03 (1.36)	1.04	–0.33	0.38	0.53	
9	2.71 (1.46)	0.27	–1.27	0.53	0.68	
2	2.30 (1.36)	0.77	–0.63	0.32		0.53
4	2.49 (1.42)	0.41	–1.20	0.56		0.74
5	3.19 (1.60)	–0.22	–1.52	0.34		0.47
8	2.70 (1.58)	0.34	–1.42	0.48		0.54

**LMA, Learning Math Anxiety; EMA, Evaluation Math Anxiety.**

#### Dimensionality

The original factor structure was tested by CFA employing the Mean-Adjusted Maximum Likelihood (MLM) estimator (Mplus software; Muthén and Muthén, 2004). This estimator provides the Satorra– Bentler Scaled chi-square (SBχ^2^; Satorra and Bentler, 2001), an adjusted and robust measure of fit for non-normal sample data. This is more accurate than the ordinary chi-square statistic (Bentler and Dudgeon, 1996). Criteria for assessing overall model fit were mainly based on practical fit measures: the ratio of chi-square to its degrees of freedom (SBχ^2^/df), the Comparative Fit Index (CFI; Bentler, 1990), the Tucker–Lewis Index (TLI; Tucker and Lewis, 1973), and the Root Mean Square Error of Approximation (RMSEA; Steiger and Lind, 1980). For the SBχ^2^/df, values of less than 3 were considered to reflect a fair fit (Kline, 2010). We deemed CFI and TLI values of 0.90 and above a fair fit (Bentler, 1995). For RMSEA, values equal to or less than 0.08 were considered to represent adequate fit (Browne and Cudeck, 1993). Results showed that goodness of fit indices for the two-factor model were all adequate (SBχ^2^ = 41.67, *df*26, *p* < 0.05, SBχ^2^/df 1.6; CFI = 0.93; TLI = 0.90; RMSEA = 0.06). Standardized factor loadings ranged from 0.45 to 0.74, all significant at the 0.001 level, just as the correlation between the two factors (0.67) (Table 2).

#### Reliability and Validity

With regard to reliability, the omega for the EES-AMAS was 0.76; 0.72 for the Learning Math Anxiety subscale (LMA), and 0.70 for the Evaluation Math Anxiety subscale (EMA) (see Supplementary Table S1 for the other reliability coefficients). All item-corrected total correlations were above 0.32 (Table 2). Concerning validity, there was a negative correlation between MA and math achievement (–0.21; *p* < 0.01).

#### Invariance Across Genders and Gender Differences

A multi-group analysis was conducted to investigate the gender invariance property of the EES-AMAS. It is a step-by-step procedure in which a series of nested models are organized in a hierarchical order. In line with the recommended practice for testing measurement invariance (Little, 1997; Vandenberg and Lance, 2000; Dimitrov, 2010), first the independence model was fitted (*SBχ*^2^ = 344.03, *df* = 72, *p* < 0.001). As reported in Table 3, the starting point was an unconstrained model to test configural invariance, which was used as a baseline for testing weak or metric factorial invariance. Criteria for assessing the difference between the competing models were based on the scaled difference chi-square test (Satorra and Bentler, 2010). Therefore, Model 1 was compared to Model 2. SBΔχ^2^ was not significant (*SBΔχ^2^*_Model 1 – Model 2_ = 9.76, *p* = 0.203), confirming that the factor loadings were equal across genders. Then, the equivalence of structural variances and covariances, which were constrained to be invariant across groups, were also tested (*SBΔχ*^2^_Model 2 – Model 3_ = 4.28, *p* = 0.233). Finally, taking Model 3 as a reference, the error variances/covariances hypothesis was tested, including constraints in error variances (Model 4). SBΔχ^2^ was not significant when comparing the two models (*SBΔχ*^2^_Model 4 – Model 5_ = 8.65, *p* = 0.470) indicating the equality of measurement errors across gender.

**TABLE 3:** Goodness-of-fit statistics for each level of structural and measurement invariance across genders.

**Model**	**SBχ^2^ (*df*)**	**SBχ^2^ /(*df*)**	***CFI***	***RMSEA***	**Model comparison**	**SBΔχ^2^**	**Δ*df***	***p***
1. Invariance of model configuration	98.70 (52)	1.9	0.90	0.08	*–*	–	–	–
2. Invariance of factor loadings	108.46 (59)	1.8	0.90	0.08	Model_1_-Model_2_	9.76	7	0.203
3. Invariance of structural variances/covariances	112.74 (62)	1.8	0.90	0.07	Model_2_-Model_3_	4.28	3	0.233
4. Invariance of measurement error	121.39 (71)	1.7	0.90	0.07	Model_3_ -Model_4_	8.65	9	0.470

**SBχ^2^, chi square test; df, degrees of freedom; CFI, comparative fit index; RMSEA, root mean square error of approximation; SBΔχ^2^, Satorra–Bentler scaled difference; Δdf, difference in degrees of freedom between nested models; p, probability value of SBΔχ^2^*-*test.**

**FIGURE 2:**
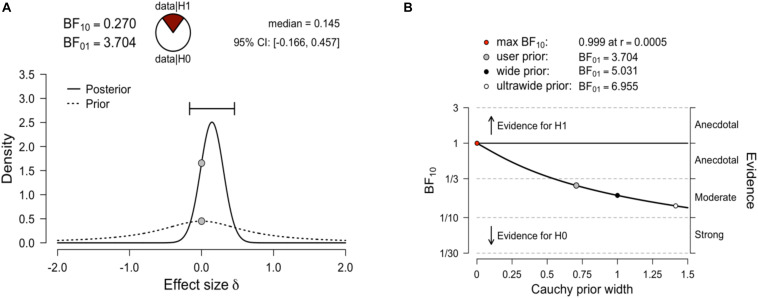
**(A)** Bayesian independent samples *t-*test for the effect size δ. The dashed line illustrates the prior distribution (default Cauchy prior centered on zero, *r* = 0.707), the solid line shows the posterior distribution. The two gray dots indicate the prior and posterior density at the test value. The probability wheel on top visualizes the evidence that the data provide for the null hypothesis (H0: effect sizes are equal) and the alternative hypothesis (auburn, H1: effect sizes are different). The median and the 95% central credible interval of the posterior distribution are shown in the top right corner. **(B)** The Bayes factor robustness plot. The plot indicates the Bayes factor BF01 (in favor of the null hypothesis) for the default prior (*r* = 0.707), a wide prior (*r* = 1), and an ultrawide prior (*r* = 1.414). All priors suggest moderate evidence for the null hypothesis, which is relatively stable across a wide range of prior distributions. Plots taken from JASP.

Having preliminarily verified the measurement equivalence of the scale across genders, we tested gender differences using the traditional frequentist approach, and also a Bayesian approach. With the traditional frequentist approach, we compared the total score (Mean _male_ = 22.47, SD _male_ = 8.4; Mean _female_ = 21.25, SD _female_ = 7.1) and the scores on each subscale (Learning: Mean _male_ = 11.91, SD _male_ = 5.5; Mean _female_ = 10.47, SD _female_ = 4.3; Evaluation: Mean _male_ = 10.56, SD _male_ = 4.2; Mean _female_ = 10.78, SD _female_ = 4.3). The results showed no significant difference between genders. Using a Bayesian approach makes it clear when a set of observed data is more consistent with the null hypothesis than the alternative. A Bayesian independent samples *t-*test was conducted using the default Cauchy prior centered on zero and with *r* = 0.707 (Ly et al., 2016). We conducted this analysis using JASP (JASP Team, 2018). The corresponding Bayes factor for the total score was 3.70 in favor of H0 over the two-sided H1. This indicated that the observed data are 3.71 times more likely under Ho than under H1. All priors suggested moderate evidence for the null hypothesis (i.e., no gender difference in MA), which was relatively stable across a wide range of prior distributions (Figure 2).

Considering the subscale scores as dependent measures, the results showed a BF01 = 1.30 for the Learning subscale and a BF01 = 5.39 for the Evaluation subscale (Supplementary Figures S1, S2). Bayes factors between 1 and 3 are considered weak evidence for the Ho (a BF value of 1 would mean that the H0 and H1 are equally likely), and values between 3 and 10 are considered to indicate moderately strong evidence. Overall, these results suggested no gender differences in math anxiety in this age group, although the evidence was somewhat weaker in the case of the Learning subscale.

### Discussion

The EES-AMAS was developed in response to the need for a brief and age-appropriate scale to assess MA in early elementary school students. The first aim of this study was to measure the factor structure of the EES-AMAS using a confirmatory procedure. The confirmatory factor analysis provided evidence of the underlying two-factor structure in younger students. Fit indices were good, and the items loaded highly on the expected factors, suggesting that the two dimensions established in the original AMAS (Learning Math Anxiety and Math Evaluation Anxiety) were evident also in the *early elementary school student* version.

Establishing the factor structure of mathematics anxiety may help with determining at this age whether anxiety pertains to the performance of mathematics in itself or whether anxiety is more related to test situations. Identifying for each student which aspect of MA is higher is also important for designing interventions. Another advantage of the EES-AMAS is its shortness. The administration time is less than 10 min and therefore, in addition to studies focusing primarily on math anxiety, it is also appropriate for multivariate studies in which many tests and scales need to be administered together. Indeed, it is useful to have a short scale. Nevertheless, it is important to balance the need to have a small number of items and the need to have good reliability. For this reason, we have developed the scale taking into consideration item wording and the length of the scale. The results showed good reliability for the EES- AMAS as a whole, and both subscales. Additionally, the scale presented good criterion validity, confirming that students with more severe MA performed less well in math tasks (Devine et al., 2012; Hill et al., 2016).

Finally, we tested invariance across genders (i.e., whether the test functions equivalently for males and females). Concerning gender differences in younger children, the majority of studies found evidence that there are small or non-existent gender differences in children of this age (e.g., Dowker et al., 2012; Harari et al., 2013; Ramirez et al., 2013; Jameson, 2014; Erturan and Jansen, 2015; Hill et al., 2016). However, in the case of most of these studies, a lack of measurement equivalence of the scales makes group comparisons ambiguous (Vandenberg and Lance, 2000). Indeed, the EES-AMAS, due to its gender invariance property, could be a useful tool to better investigate gender differences in young children in future studies. In the current study, we found no significant gender difference in math anxiety in our sample, either in the total math anxiety score or in the subscale scores. We conducted Bayesian analyses to quantify the evidence for the null hypothesis in each case. We found moderate evidence in favor of the null hypothesis in the case of the total score and the Evaluation subscale score. However, the evidence for no gender difference was weaker in the case of the Learning subscale. We will return to this issue in Study 2.

## Study 2

Although MA is considered a global phenomenon and it is supposed to be a transcultural trait (Ma, 1999), the majority of research on MA has been conducted in North America (cf., Morsanyi et al., 2016; Mammarella et al., 2019). One large-scale attempt to evaluate MA across different countries has been undertaken by the PISA assessment in 2012. Results showed that 33% of 15-year-old students across 65 countries who participated in this assessment reported feeling helpless when solving math problems. However, this study has only compared responses to single items, and did not investigate the structure of MA across countries. Very few studies have assessed the structure of MA in children using the same scale translated into different languages. Ho et al. (2000) tested the dimensionality of the MAQ (Wigfield and Meece, 1988) with 11 year-old children, confirming its two-dimensional structure (i.e., affective and cognitive). Indeed, the structure of MA has been found to be similar in American, Chinese and Taiwaneese students. Only the study of Wood et al. (2012) investigated the structure of MA in early elementary school students (second and third graders) in German and Brazilian samples and showed a similar structure across countries. However, even in this study, the invariance of the scale across countries has not been investigated.

In the current study, the participants were early elementary school pupils, recruited from two countries: Italy and the UK. The UK sample was from Northern Ireland, which has the youngest school starting age (4 years) among the 37 countries participating in Eurydice, the information network on education in Europe (Eurydice at NFER, 2012). In Italy, children start school at 6 years of age. We have recruited 6- and 7-year-old pupils from both countries, which made it possible to test the equivalence of the EES-MAS not only across languages, but also across educational contexts. The aim of this analysis was to test whether observed MA scores depended *only on the latent construct*, and not on group membership. Similar to Study 1, we have applied multiple group confirmatory factor analysis (MGCFA), in which the theoretical model is compared to the observed structure in two samples. Additionally, in both samples, we tested the criterion validity of the scale, measuring its relations with math achievement (as measured by a math test, and by teacher’s ratings of each child’s achievement). Based on the typical findings in the literature, we expected a small- to medium negative correlation between math anxiety and math performance. Additionally, we tested the convergent validity of the EES-AMAS by measuring its relationship with the CMAQ-R (the Child Math Anxiety Questionnaire –Revised; Ramirez et al., 2016), which has been developed for the same age group as our scale, although it is much longer. We also investigated the relationship between the EES-AMAS and children’s state anxiety after they completed the math test.

### Materials and Methods

#### Participants

The participants were 223 early elementary school students (mean age = 6.7 years; *SD* = 0.6; 47% female) 46% attending primary school in Forlì (Italy; mean age = 6.41 years; *SD* = 0.49; 40% female) and 54 % in Belfast (UK; mean age = 7.11 years; *SD* = 0.66; 52% female).

#### Materials and Procedure

The Italian version of the EES-AMAS was administered to the Italian pupils. The English version of the EES-AMAS was obtained using a forward-translation method. Two non-professional translators worked independently, and then they compared their translations with the purpose of assessing equivalence. The wording and content of the items was also discussed with schoolteachers to obtain a final version. As in Study 1, an interviewer presented individually a brief description of anxiety with some examples and participants responded to items on a pictorial scale consisting of partially filled boxes with a differing level of content, representing “little” to “much” anxiety.

The CMAQ- R (Ramirez et al., 2016) was designed to be appropriate for first and second grade children. It contains 16 items that ask children how nervous they would feel during various math-related situations. Responses are collected using a 5-point Likert scale ranging from 1 (not nervous at all) to 5 (very, very nervous), which are represented in the form of smiley faces. Children have to respond by pointing at the appropriate smiley face on the scale. High scores on the scale indicate high math anxiety. The Italian version of the CMAQ-R was obtained from the English version using a forward-translation method. Two non-professional translators worked independently, and then they compared their translations with the purpose of assessing equivalence. With regard to reliability, the internal consistency Cronbach’s alpha for the CMAQ-R was 0.83 (CI 0.82–0.87) in the Italian sample and 0.80 (CI 0.74–0.85) in the British sample.

State math anxiety was measured by a single-item scale, which was administered to pupils after they completed the math test. The same smiley face scale was used as in the CMAQ-R (Ramirez et al., 2016). Children were asked to point to one of five smiley faces to indicate how nervous they felt about completing the math problems. The face on the leftmost side indicated that the child was not nervous at all, whilst the face on the rightmost side indicated that the child felt very, very nervous.

Math Performance in both the Italian and the UK sample was measured by a test developed for the purposes of this study. The two tests were developed using the same criteria, but were different in their contents due to the fact that children at age 6 attend the first primary school grade in the Italian school system, and the third grade in Northern Ireland. In detail, the UK test was based on items from the Test of Early Mathematics Ability (TEMA-3; Ginsburg and Baroody, 2003). The test consisted of 38 items, which were administered in a single session in four parts, with short breaks in between. The tasks were read out to children to minimize the effect of reading ability on children’s performance. The items covered addition and subtraction problems including both single- and two-digit numbers, additions and subtractions with multiples of ten, and word problems that also relied on simple addition or subtraction procedures. The items were selected from a set of 50 problems, which were piloted in a separate sample of 27 children. Tasks with accuracy levels between 35 and 75% were retained to ensure a good variability of scores on the test. The same procedure was adopted to develop the test administered to the Italian sample. In the pilot phase, a set of 50 math tasks were used that included addition and subtraction with both single- and two-digit numbers, additions and subtractions with multiples of ten, word problems relying on addition and subtraction, and number sequencing. These tasks were administered to a sample of 37 children. Nineteen items with accuracy levels between 35 and 75% were retained for the final test, including 5 additions, 4 subtractions, 5 word problems with addition, 3 word problems with subtractions, and 2 number sequencing tasks. A single composite score, based on the sum of correct responses, was calculated for both samples. Cronbach’s alpha was 0.92 in the UK sample, and 0.86 in the Italian sample.

Teachers were also asked to provide a rating of each child’s math achievement using a 5-point scale: 1 = working well below the expected level of attainment for his/her age; 2 = working below the expected level of attainment for his/her age; 3 = working toward the expected level of attainment for his/her age; 4 = working within the expected level of attainment for his/her age; 5 = working beyond the expected level of attainment for his/her age.

The study was approved by the School of Psychology ethics committee at Queen’s University Belfast (UK), and by the ethics committee of the University of Bologna (Italy). Informed consent was gained from parents prior to their child’s participation, whilst assent was obtained from the children before they took part in the study. Children were tested in two sessions: in the first session, they were tested in groups of 4–8 in their classes, and they completed the math assessment. The tasks were administered in four parts, with short breaks in between. At the end of the session, children were asked to say how nervous they felt while completing the math tasks. Individual sessions were carried out at least 1 day after the group session and involved children completing the math anxiety questionnaires. The scales were administered in a fixed order with the CMAQ-R always administered first. The reason for this was that the EES-AMAS included detailed instructions, which might have affected responses on the CMAQ-R. Teachers provided ratings of each child’s math achievement in their own time.

### Results

First, as a prerequisite, the baseline model was tested separately for each country. For the Italian sample, the two-factor model had goodness of fit indices as follows: SBχ^2^/*df* = 1.55, TLI = 0.90, CFI = 0.92; and RMSEA 0.07. Standardized factor loadings ranged from 0.43 to 0.74, all significant at the 0.001 level, just as the correlation between the two factors (0.77). For the British sample, the two-factor model was associated with the following goodness of fit indices: SBχ^2^/*df* = 1.45, TLI = 0.90, CFI = 0.91; RMSEA.07. Standardized factor loadings ranged from 0.37 to 0.70, all significant at the 0.001 level, as well as the correlation between the two factors (0.75) (Table 4).

**TABLE 4:** Means, standard deviations (SDs), item- total correlation for each item and factor loadings of the EES-AMAS for each sample.

	**Italian sample**				**British sample**			
**Item**	**Mean (*SD*)**	**Corrected item-total correlations**	**LMA**	**EMA**	**Mean (*SD*)**	**Corrected item-total correlations**	**LMA**	**EMA**
1	1.80 (1.38)	0.47	0.56		1.19 (0.67)	0.30	0.37	
3	1.88 (1.29)	0.41	0.47		1.82 (1.45)	0.52	0.70	
6	1.87 (1.31)	0.44	0.57		1.84 (1.95)	0.33	0.50	
7	2.02 (1.41)	0.38	0.43		1.66 (1.07)	0.38	0.48	
9	2.52 (1.61)	0.61	0.74		2.57 (1.45)	0.44	0.41	
2	2.53 (1.49)	0.59		0.69	2.81 (1.44)	0.46		0.58
4	2.26 (1.34)	0.46		0.56	3.01 (1.49)	0.45		0.65
5	3.50 (1.40)	0.49		0.63	3.29 (1.53)	0.36		0.45
8	2.61 (1.40)	0.45		0.60	2.43 (1.40)	0.53		0.62

#### Invariance Across Languages/Educational Contexts

A multi-group analysis was conducted to investigate the cross-language invariance property of the EES-AMAS. It is a step-by-step procedure in which a series of nested models are organized in a hierarchical order. In line with the recommended practice for testing measurement invariance (Little, 1997; Vandenberg and Lance, 2000; Dimitrov, 2010) first the independence model was fitted (SBχ^2^ = 540.38, *df* = 72, *p* ≤ 0.001). As reported in Table 5, the starting point was an unconstrained model to test configural invariance, which was used as a baseline for testing weak or metric factorial invariance. Criteria for assessing the difference between the competing models were based on the scaled difference chi-square test (Satorra and Bentler, 2010). Therefore, Model 1 was compared to Model 2 and SBΔχ^2^ was not significant (SBΔχ^2^_Model 1 – Model 2_ = 13.06, *p* = 0.071), confirming that the factor loadings were equal across languages. Then, the equivalence of structural variances and covariances which were constrained to be invariant across groups, were also tested (SBΔχ^2^_Model 2 – Model 3_ = 1.40, *p* = 0.703). Finally, taking Model 3 as a reference, the error variances/covariances hypothesis was tested including constraints in error variances (Model 4). SBΔχ2 was not significant when comparing the two models (SBΔχ^2^_Model 4 – Model 5_ = 8.31, *p* = 0.503), indicating the equality of measurement errors across languages.

**TABLE 5:** Goodness-of-fit statistics for each level of structural and measurement invariance across languages.

**Model**	**SBχ^2^ (*df*)**	**SBχ^2^ /(*df*)**	***CFI***	***RMSEA***	**Model comparison**	**SBΔχ^2^**	**Δ*df***	***p***
1. Invariance of model configuration	109.57 (52)	2.1	0.90	0.07	–	–	–	–
2. Invariance of factor loadings	122.63 (59)	2.1	0.90	0.07	Model_1_-Model_2_	13.06	7	0.071
3. Invariance of structural variances/covariances	124.03 (62)	2.0	0.89	0.07	Model_2_-Model_3_	1.40	3	0.704
4. Invariance of measurement error	132.34 (71)	1.9	0.89	0.06	Model_3_ -Model_4_	8.31	9	0.503

**SBχ^2^, chi square test; df, degrees of freedom; CFI, comparative fit index; RMSEA, root mean square error of approximation; SBΔχ^2^, Satorra–Bentler scaled difference; Δdf, difference in degrees of freedom between nested models; p, probability value of SBΔχ^2^*-*test.**

Having verified the measurement equivalence of the scale, we tested group differences in mean scores on the total score (Mean_Italian_ = 21.01, SD _Italian_ = 7.7; Mean_British_ = 20.59, SD_British_ = 6.6), and each subscale (Learning: Mean_Italian_ = 10.9, SD _Italian_ = 4.7; Mean_British_ = 9.1, SD_British_ = 3.5; Evaluation: Mean_Italian_ = 10.9, SD _Italian_ = 4.1; Mean_British_ = 11.5, SD _female_ = 4.0). Results showed no significant differences between the groups, indicating that, at 6-years of age, Italian and Northern Irish children experienced similar levels of math anxiety.^2^

**FIGURE 3:**
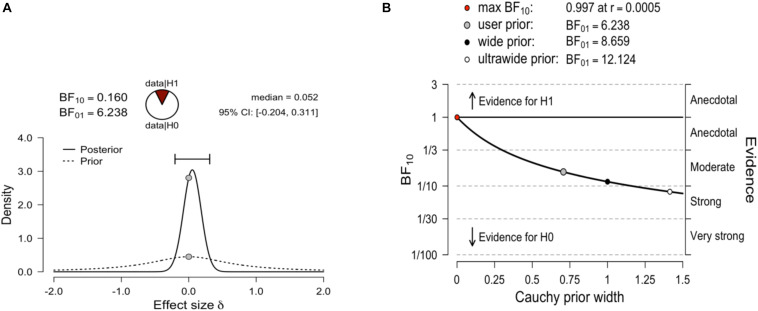
**(A)** Bayesian independent samples *t-*test for the effect size δ. The probability wheel on top visualizes the evidence that the data provide for the null hypothesis (H0: effect sizes are equal) and the alternative hypothesis (auburn, H1: effect sizes are different). The median and the 95% central credible interval of the posterior distribution are shown in the top right corner. **(B)** The Bayes factor robustness plot. The plot indicates the Bayes factor BF01 (in favor of the null hypothesis) for the default prior (*r* = 0.707), a wide prior (*r* = 1), and an ultrawide prior (*r* = 1.414).

A Bayesian independent samples *t-*test was conducted using the default Cauchy prior centered on zero and with *r* = 0.707. The corresponding Bayes factor for the total score was 6.23 in favor of H0 over the two-sided H1. All priors suggest moderate evidence for the null hypothesis, which is relatively stable across a wide range of prior distributions (Figure 3).

Considering the subscale scores as dependent measures, the results showed a BF01 = 1.38 for the Learning subscale, indicating weak evidence in favor of the null hypothesis. In the case of the Evaluation subscale, a Bayesian independent samples *t*-test (BF01 = 3.74) indicated moderate evidence in favor of the null hypothesis (Supplementary Figures S3, S4).

#### Reliability and Validity

With regard to reliability, in the Italian sample omega was 0.79 and in the English sample it was 0.74. In both samples, all item-corrected total correlations were above 0.30 (Table 4).

Concerning validity, to investigate the relationship between MA and math achievement, correlations between the EES-AMAS and math test scores, as well as teacher’s ratings of children’s math achievement were calculated. The results showed that higher levels of MA were associated with poorer math performance in both samples, and the strength of this relationship was moderate (Table 6).

**TABLE 6:** Descriptive statistics for the measures, and correlations between the measures of math achievement and math anxiety (results for the UK sample are presented in brackets).

	**EES-AMAS**	**CMAQ-R**	**State math anxiety**	**Math test performance**	**Math achievement (teacher rating)**
EES-AMAS	–				
CMAQ-R	0.70**(0.69**)	–			
State math anxiety	−0.04(0.22*)	0.09(0.23*)	–		
Math test performance	−0.38**(−0.32**)	−0.39**(−0.43**)	−0.04(−0.21*)	–	
Math achievement (teacher rating)	−0.30**(−0.34**)	−0.32**(−0.29**)	−0.09(−0.24**)	0.53**(0.70**)	–
*M (SD)* Italian	21.01 (7.75)	39.16 (11.68)	1.69 (1.20)	21.83 (8.80)	3.54 (1.01)
*M (SD)* UK	20.59 (6.59)	35.69 (10.96)	1.83 (1.44)	22.47 (8.42)	3.52 (1.11)

* p < 0.05; ** p < 0.01.

To analyze convergent validity, we tested the relationship between the EES-AMAS and the CMAQ-R, as well as children’s state anxiety immediately after completing a math test. Strong, positive correlations were found in both samples between the two MA scales. Regarding state math anxiety, there was no relationship between trait and state math anxiety in the Italian sample, but in the UK sample there was a weak positive correlation.

Similar to Study 1, we checked whether there were any gender differences in MA. Additionally, we also made comparisons between girls’ and boys’ math performance based on their math test scores and teacher’s ratings (Table 7). There were no gender differences in MA either in the Italian or in the UK sample (*p*_s_> 0.40). This was also the case when we checked separately whether there were gender differences in Learning or Evaluation MA. There were also no gender differences in math performance, although in the Italian sample, there was a non-significant trend toward boys scoring higher on the math test (*p* = 0.075).

**TABLE 7:** Gender differences in math anxiety and math performance.

		**Male *M* (S*D*)**	**Female *M* (*SD*)**	***t* (*df*)**	***p***	**BF_01_**
EES-AMAS	Italy	20.73 (7.72)	21.44 (7.86)	−0.456(101)	0.650	4.30
	UK	21.12 (6.54)	20.11 (6.65)	0.839 (118)	0.403	3.74
Learning anxiety	Italy	9.87 (4.59)	10.44 (4.98)	−0.595(101)	0.553	4.03
	UK	9.28 (3.88)	8.89 (3.20)	0.606 (118)	0.546	4.35
Evaluation anxiety	Italy	10.85 (4.26)	11.00 (3.99)	−0.174(101)	0.863	4.65
	UK	11.86 (3.77)	11.24 (4.29)	0.839 (118)	0.403	3.74
Math test performance	Italy	23.08 (9.19)	19.87 (7.87)	1.799 (98)	0.075	1.12
	UK	23.46 (7.68)	21.59 (8.99)	1.217 (118)	0.226	2.63
Math achievement (teacher rating)	Italy	3.50 (1.14)	3.60 (.78)	−0.486(100)	0.628	4.22
	UK	3.53 (1.17)	3.51 (1.06)	0.090 (118)	0.928	5.11

Bayesian independent samples *t*-tests were conducted for the effect size δ (Table 7). The results indicated moderate evidence in favor of the null hypothesis considering gender as the independent variable in each country.

### Discussion

Study 2 tested the equivalence of the Italian and English versions of the EES-AMAS, attesting the appropriateness of the scale to be used in both languages and educational contexts. The equivalence of the scale across countries is important for being able to generalize findings obtained with one country/language version of the test to other countries.

Additionally, we tested the validity of the scale in both populations. In particular, we have tested the criterion validity of the scale, using teacher ratings and a math test adapted for both countries. As expected, MA negatively correlated with the measures of math achievement in both countries. Moreover, the strength of this relationship was moderate. This is an important finding, because some previous studies did not find a relationship between math achievement and math performance in young pupils (Cain-Caston, 1993; Thomas and Dowker, 2000; Krinzinger et al., 2009; Dowker et al., 2012). Nevertheless, in line with our findings, other studies have reported a relationship between MA and math performance even in the first school grades (Wu et al., 2012, 2014; Ramirez et al., 2013; Vukovic et al., 2013; Ramirez et al., 2016). It has also been argued that young children generally have positive feelings about mathematics, but their feelings and attitudes deteriorate with age (Wigfield and Meece, 1988; Ma and Kishor, 1997). Related to this point, our findings show that young pupils in both countries tended to report low levels of anxiety (as indicated by their ratings of the scale items). Additionally, similar to Study 1, there were no gender differences in MA in either the Italian or the UK sample in the case of the total score, and no gender difference in either the Learning or the Evaluation subscale, with moderate evidence for the null hypothesis in both samples.

We also investigated the validity of the EES-AMAS by assessing its relationship with a well-known measure of MA developed for this age group, the CMAQ-R. The strong, positive correlation between the two measures confirmed that the two scales measured the same construct. We have also measured the relations between the EES-AMAS and children’s self-reported state math anxiety after completing the math test. We have found a weak positive correlation between state and trait anxiety in the UK sample. However, in the Italian sample, there was no relationship between state and trait anxiety. Additionally, although the CMAQ-R is much longer, the two MA scales showed very similar relations with math performance.

A limitation of this study is that the math assessment was developed specifically for this study, and therefore its validity has not been independently established. However, the math test was based on items from a standardized, curriculum-based test, the TEMA-3, and it had high internal consistency in both samples. We also piloted the test in a separate sample of children in both countries to make sure that the items covered a range of difficulty levels, although very easy or very difficult items were not included. Another limitation is that we used an *ad hoc*, single item scale to measure state math anxiety. Although state math anxiety was related to MA and math performance in the UK sample, no similar relations were found in the Italian group. Given that this measure has not been used outside this study, these findings are difficult to interpret.

## Conclusion

MA is a widespread, worldwide problem affecting all age groups. Recent studies have shown that MA affects performance even in the first years of education (Harari et al., 2013; Ramirez et al., 2013). However, to date there are only a few studies that have investigated MA in this age group. One of the problems which contributes to the difficulty of conducting research into MA in young children relates to the question of how MA should be measured in this age group. Based on our review of the psychometric properties (i.e., dimensionality, validity and reliability) of the scales developed for this age group, we have identified areas for improvement in the assessment of MA in young children. In order to address these limitations, the current study aimed at developing a new instrument to assess MA in early elementary school students.

Among the existing measures of MA, the AMAS (Hopko et al., 2003) has been used with adults in different cultural and linguistic contexts, and it showed good psychometric properties. Additionally, it was adapted for primary school children from 8 years of age, and was shown to be a valid and reliable scale for measuring MA in children (Carey et al., 2017; Caviola et al., 2017). For all these reasons, the AMAS was chosen as our starting point to develop the *Early Elementary School Students – Abbreviated Math Anxiety Scale* (EES-AMAS). Although the EES-AMAS is a short scale (similar to the original AMAS), it showed good validity and reliability, and also maintained the two-factors structure of the original scale, indicating, that from a young age, children experience anxiety (even if it is not too intense) in both math learning and evaluation contexts. Given that the same factors appear to underlie MA in the case of younger and older children, adolescents and adults, it might be possible for future studies to longitudinally track the developmental trajectories of these factors. Indeed, currently very little is known about how MA within the same individual unfolds over time, and there is especially little understanding of the early origins of MA.

The new scale was shown to be invariant across genders and linguistic/educational contexts. Although we have only tested the equivalence of the scale across two countries, the evidence for equivalence is a promising initial result, given the differences between the school systems in Italy and Northern Ireland (most notably, there is a 2-year difference in children’s school starting age).

Using our new scale, we have found no evidence of gender differences in MA, with Bayesian *t*-tests indicating moderate evidence in favour of the null hypothesis. This finding was consistent across all samples of children (two from Italy and one from the UK) that were included in our studies. This is an important result given the ubiquitous evidence for gender differences in studies with older age groups. This finding also suggests that gender differences in MA are unlikely to have a biological basis, and most likely reflect societal influences, and differences in the experiences of male and female pupils both within and outside of the educational context.

A novelty of our scale is that we have introduced a pictorial rating scale, consisting of partially filled boxes, which was easy to use for children even at this young age, and avoided the problems associated with other rating scales. In particular, when a rating scale consisting of smiley and sad faces is used, young children might be inclined to select faces that they find more attractive instead of selecting a face that best represents their emotional state.

In recent years, the assessment of MA has attracted increasing research attention, and several studies have focussed on young children. Nevertheless, the instruments used in these studies had various shortcomings. The EES-AMAS is a psychometrically sound short scale, which offers several advantages over previously developed scales. Indeed, with the advancement of knowledge about MA, and research questions becoming increasingly complex and involving a growing number of constructs, shorter scales offer added value (Ziegler et al., 2014). The EES-AMAS can be used to investigate the development of MA, as well as to further investigate the presence or absence of gender differences in MA in young children. If the invariance of the scale is further confirmed across different countries and languages, it could also offer support for the claim that the MA construct generalizes across countries, and linguistic and educational contexts. Finally, future studies could also investigate potential differences in the two dimensions of MA (i.e., Evaluation and Learning MA) across countries. Indeed, countries differ in the age at which various forms of assessment are introduced, and in the ways children are given feedback on their performance, which might lead to differences in the development of MA.

## Data Availability Statement

The datasets generated for this study are available on request to the corresponding author.

## Ethics Statement

The studies involving human participants were reviewed and approved by the study was approved by the School of Psychology ethics committee at Queen’s University Belfast (UK), and by the ethics committee of the University of Bologna and University of Florence (Italy). Written informed consent to participate in this study was provided by the participants’ legal guardian/next of kin.

## Author Contributions

CP, KM, and CT conceived the study. CP and MD did the analyses. CP and KM wrote the manuscript. VI, VG, and PO’C collected the data. All authors discussed the results together and contributed to the final manuscript, doing critical revisions and giving suggestions, read the manuscript, and approved the submitted version.

## Conflict of Interest

The authors declare that the research was conducted in the absence of any commercial or financial relationships that could be construed as a potential conflict of interest.

## Figures and Tables

**Table:** 

	**A little anxiety**				**Much anxiety**
1. When you are using the number line.	1	2	3	4	5
2. When you think about a maths test that you have to do soon.	1	2	3	4	5
3. When you watch your teacher solving a maths sum on the whiteboard.	1	2	3	4	5
4. When you are taking a maths test at school.	1	2	3	4	5
5. When your maths teacher gives you homework that is long and difficult.	1	2	3	4	5
6. When your maths teacher explains a new topic.	1	2	3	4	5
7. When another student solves a sum on the whiteboard.	1	2	3	4	5
8. When your maths teacher asks you to solve a maths sum.	1	2	3	4	5
9. When you have to learn how to solve a new kind of maths sum.	1	2	3	4	5

## Footnotes

1 These tests did not have exactly the same items, because the children in the two countries attended different school grades. Nevertheless, the types of items were very similar, as well as the overall structure of the assessment and the way the tests were administered.

2 It is of note that the CMAQ-R indicated a significant difference in MA across countries [*t*(221) = 2.28 *p* = 0.023]. Nevertheless, given that the measurement equivalence of the CMAQ-R across countries has not been verified, it is possible that this difference was the result of different interpretation of the items by Italian and British children.

## Appendix: The Early Elementary School Abbreviated Math Anxiety Scale

Now i am going to read you some sentences about situations that can happen at school. For each sentence you should tell me how much anxious you feel.

Do you know what anxiety is?

Feeling anxious means that you feel worried, upset, your hands sweat, you are afraid to give the wrong answer.

For example, if your teacher asks you something, how do you feel? do you feel anxious? Are you worried? are your hands sweating? Are you afraid to give your teacher the wrong answer?

To tell me how anxious you feel in each situation, you should point at one of these squares:

If you choose the one on your left, that is almost empty, it means that you feel just a little anxious in that situation. if you choose the one on your right, that is completely filled, it means that you feel really anxious in that situation (explain the other squares).

Do you understand? let’s begin.

**How much anxiety do you feel:**
