# Supplementary material for: The Early Elementary School Abbreviated Math Anxiety Scale (the EES-AMAS): A New Adapted Version of the AMAS to Measure Math Anxiety in Young Children
Source: Front Psychol. 2020 May 21;11:1014. doi: 10.3389/fpsyg.2020.01014 (PMC7253683; doi:10.3389/fpsyg.2020.01014)
Supplement: Supplementary file 1 [file Data_Sheet_1.docx]

**Supplementary Table S1**

Reliability coefficients (omega, alfa and ordinal alfa) for each subscale and total scale in each study

|  | Omega | Alfa | Ordinal Alfa |
| --- | --- | --- | --- |
| Study 1 |  |  |  |
| LMA | .72 | .71 | .78 |
| EMA | .70 | .66 | .71 |
| EES-AMAS | .76 | .76 | .78 |
| Study 2 |  |  |  |
| *Italian sample* |  |  |  |
| LMA | .70 | .70 | .79 |
| EMA | .72 | .71 | .76 |
| EES-AMAS | .79 | .79 | .83 |
| *British sample* |  |  |  |
| LMA | .63 | .60 | .74 |
| EMA | .64 | .63 | .66 |
| EES-AMAS | .74 | .74 | .78 |
|  |  |  |  |
