# Supplementary material for: The Early Elementary School Abbreviated Math Anxiety Scale (the EES-AMAS): A New Adapted Version of the AMAS to Measure Math Anxiety in Young Children
Source: Front Psychol. 2020 May 21;11:1014. doi: 10.3389/fpsyg.2020.01014 (PMC7253683; doi:10.3389/fpsyg.2020.01014)

**Supplementary Figure S1**

Bayesian independent samples *t* test with *gender* as an independent variable and with scores on the *Learning subscale* as a dependent measure

 
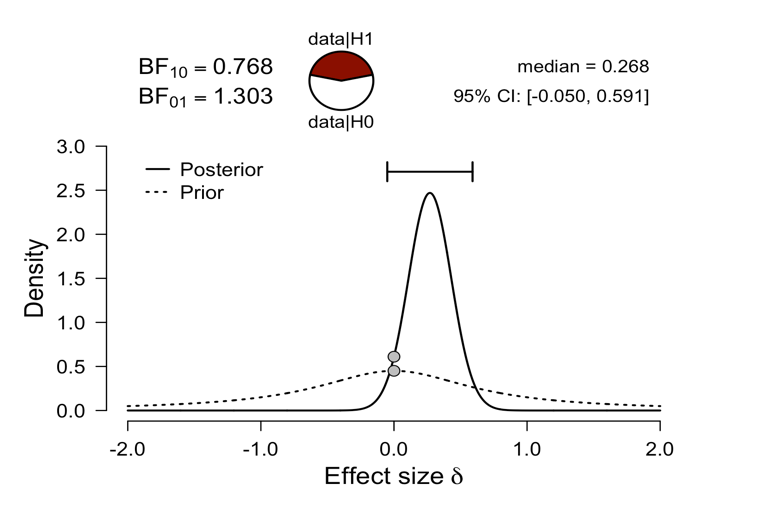

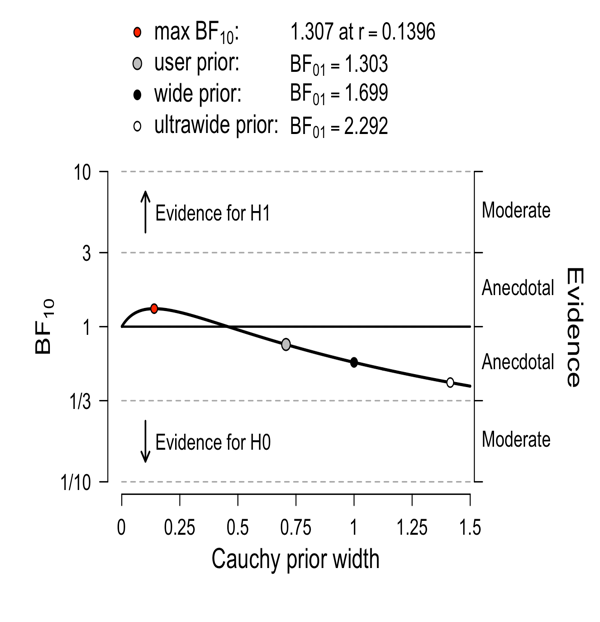


**Supplementary Figure S2**

Bayesian independent samples *t* test with *gender* as an independent variable and scores on the *Evaluation subscale* as a dependent measure


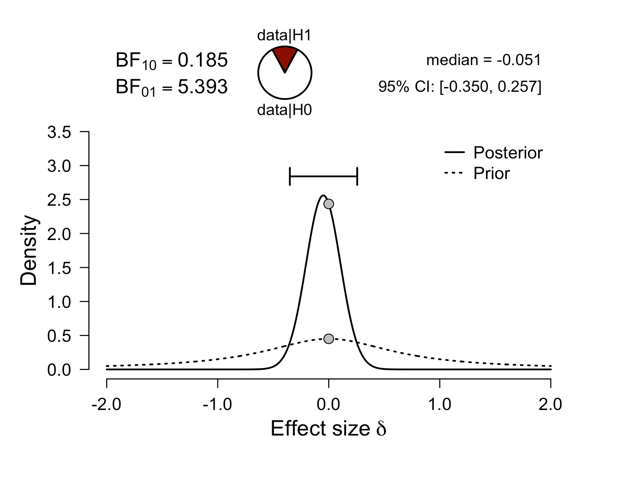

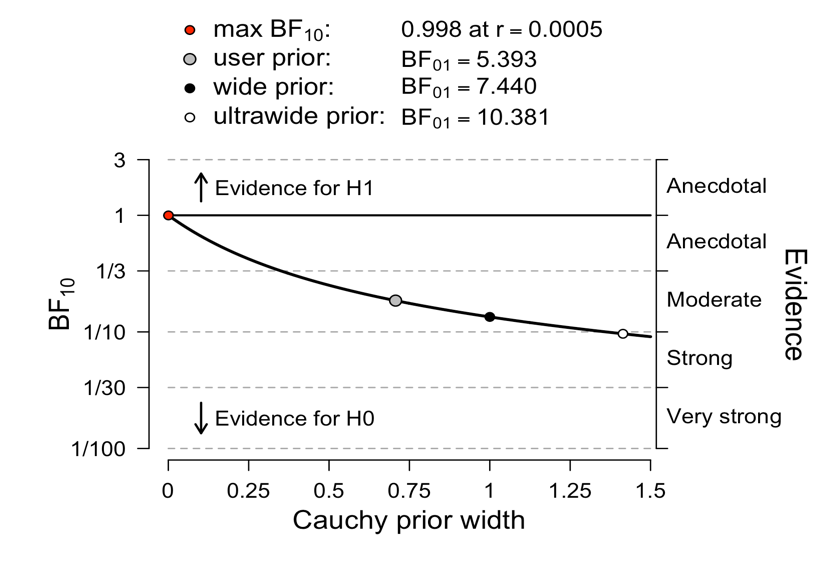

Supplement: Supplementary file 2 [file Data_Sheet_2.docx]
