# Supplementary material for: The Early Elementary School Abbreviated Math Anxiety Scale (the EES-AMAS): A New Adapted Version of the AMAS to Measure Math Anxiety in Young Children
Source: Front Psychol. 2020 May 21;11:1014. doi: 10.3389/fpsyg.2020.01014 (PMC7253683; doi:10.3389/fpsyg.2020.01014)

**Supplementary Figure S3**

Bayesian independent samples *t* test with *country* as an independent variable and scores on the *Learning subscale* as a dependent measure


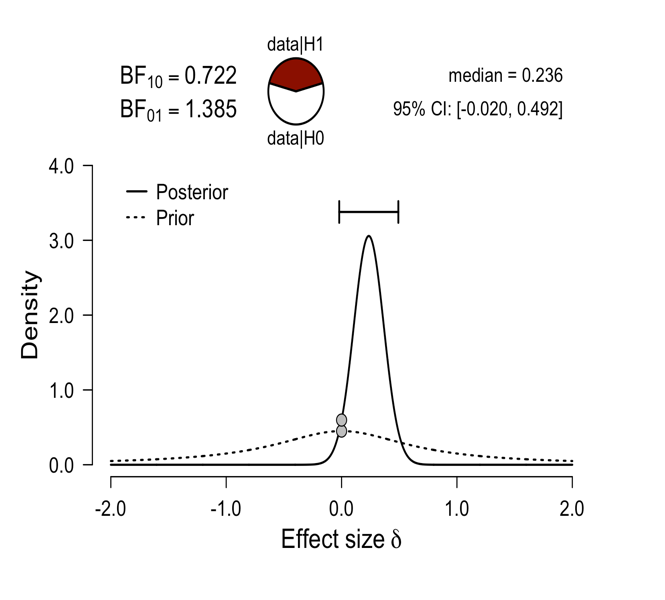

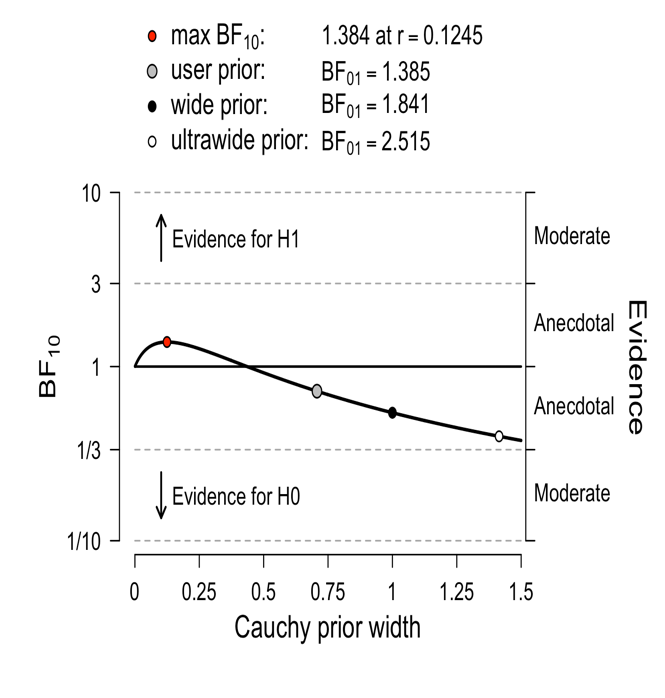


**Supplementary Figure S4**

Bayesian independent samples *t* test with *country* as an independent variable and scores on the *Evaluation subscale* as a dependent measure


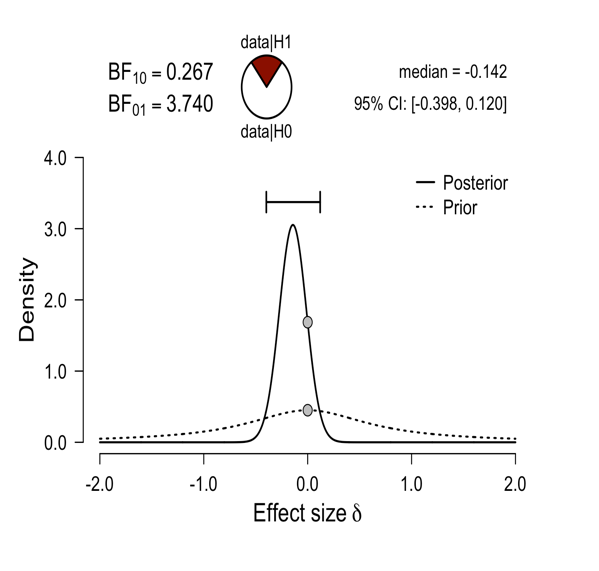

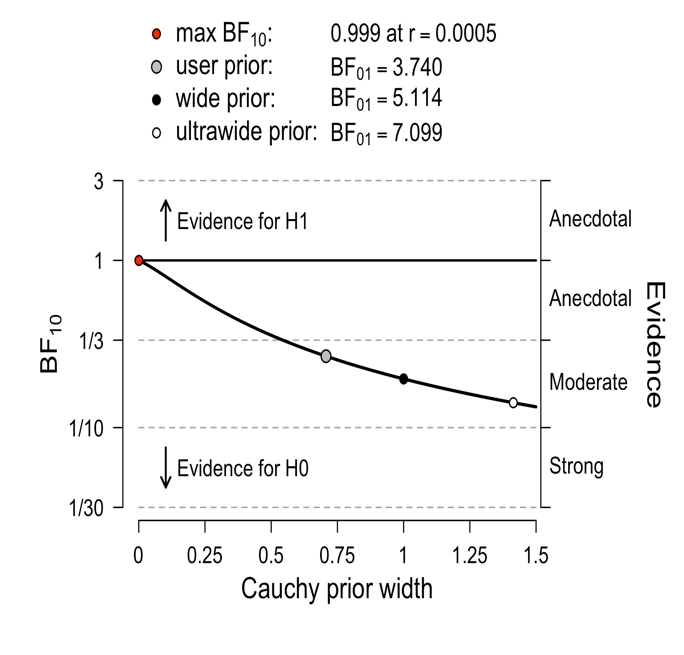

Supplement: Supplementary file 3 [file Data_Sheet_3.docx]
